# A transparent, solvent-free laminated top electrode for perovskite solar cells

**DOI:** 10.1080/14686996.2016.1176512

**Published:** 2016-06-13

**Authors:** Mohammed Makha, Silvia Letícia Fernandes, Sandra Jenatsch, Ton Offermans, Jürg Schleuniger, Jean-Nicolas Tisserant, Anna C. Véron, Roland Hany

**Affiliations:** ^a^ Empa, Swiss Federal Institute for Materials Science and Technology, Laboratory for Functional Polymers, Dübendorf, Switzerland; ^b^ UNESP – Universidade Estadual Paulista – Instituto de Química, POSMAT, Araraquara, Brazil; ^c^ CSEM, Centre Suisse d’Electronique et Microtechnique SA, Muttenz, Switzerland; ^d^ Nanotechnology Group, ETH Zurich, Rüschlikon, Switzerland

**Keywords:** Perovskite, solar cell, lamination, transparent electrode, 50 Energy materials, 102 Porous/Nanoporous/Nanostructured materials, 209 Solar cell/Photovoltaics

## Abstract

A simple lamination process of the top electrode for perovskite solar cells is demonstrated. The laminate electrode consists of a transparent and conductive plastic/metal mesh substrate, coated with an adhesive mixture of poly(3,4-ethylenedioxythiophene):poly(styrenesulfonate), PEDOT:PSS, and sorbitol. The laminate electrode showed a high degree of transparency of 85%. Best cell performance was achieved for laminate electrodes prepared with a sorbitol concentration of ~30 wt% per milliliter PEDOT:PSS dispersion, and using a pre-annealing temperature of 120°C for 10 min before lamination. Thereby, perovskite solar cells with stabilized power conversion efficiencies of (7.6 ± 1.0)% were obtained which corresponds to 80% of the reference devices with reflective opaque gold electrodes.

## Graphical abstract

**Figure:**
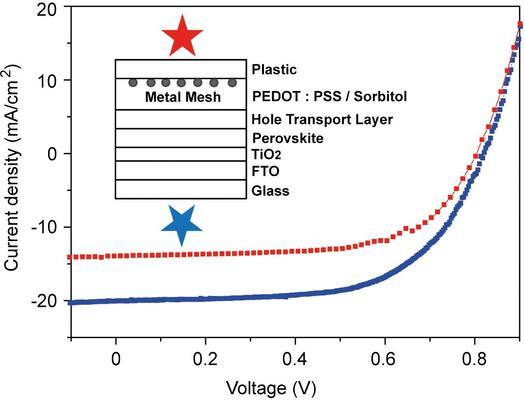

## 1. Introduction

Organic-inorganic lead halide perovskite solar cells (PSCs) are attracting interest in research and industrial laboratories due to their potential as high-performing devices for solar energy conversion.[1–3] Similar to organic solar cells (OSCs), the fabrication of PSCs is compatible with low temperatures and high-volume, large-area processing techniques from solution, so they may be developed at low-cost. Roll-to-roll compatible thin film processing techniques known from the field of organic electronics [4] are now tested to scale laboratory size PSCs into modules. PSCs have been fabricated by blade- and slot-die coating outside the glovebox under ambient conditions.[5,6] PSCs were upscaled on flexible substrates, including printing of the back electrode.[7]

In many cases, in high-performing PSCs charge collection at the counter electrode is achieved by evaporating an opaque gold metallic electrode onto the active material. Gold makes an ohmic contact but it limits the potential for solvent-based high-throughput cell fabrication. In addition, gold prevents PSCs from being used in semitransparent application. Therefore, substitution of the vacuum-deposited back electrode is an important challenge. Resorting to the concepts acquired so far for transparent and conductive electrodes in the field of optoelectronic devices,[8] several alternatives to gold as the charge-collecting top contact have been reported. Successful examples include spray-coating and mechanical transfer of silver nanowires.[9,10] Carbon nanotube and graphene electrodes were applied by a lamination process.[11–13] Similarly, thin films of poly(3,4-ethylenedioxythiophene):poly(styrenesulfonate) (PEDOT:PSS) were deposited onto the hole transporting material of PSCs by transfer-lamination.[14,15]

Fabricating two parts of a PSC separately on different substrates and finishing the device by a lamination step is an attractive and scalable technique. Thereby, the top electrode has not to be deposited directly onto the sensitive perovskite layer. The key step is that the laminated parts make intimate contact, both mechanically and electronically. Several techniques reported for OSCs used thin films of water-based PEDOT:PSS dispersions that were coated onto the active layer during lamination.[16–18] It is well known that halide perovskites are sensitive to polar solvents and humidity.[14,15] Therefore, these lamination concepts cannot be directly adopted for the fabrication of PSCs. Recently, however, organic-solvent based PEDOT was successively coated directly on the perovskite and was tested as hole-extraction layer and interlayer between the hole-transporting material and a metal grid electrode.[19–21]

PEDOT:PSS can also be laminated after drying. However, since a carefully dried and water-free PEDOT:PSS film is little tacky, an adhesive must be added, the best known being D-sorbitol. PEDOT:PSS/sorbitol films act as conductive glue and can effectively laminate various materials when heated above the melting point of sorbitol (~95°C).[22] ITO/PEDOT:PSS/sorbitol or transparent Ag network/PEDOT:PSS/sorbitol laminate electrodes were developed for the fabrication of semitransparent OSCs.[23,24] For inverted OSCs, a flexible plastic/100 nm thick Ag film/PEDOT:PSS/sorbitol laminate electrode was used.[25] Best devices were obtained when adding 10 wt% sorbitol to the PEDOT:PSS dispersion. The electrode was pre-annealed at 115°C for 10 min, and cell components were hot press laminated at 130°C.

Here, we demonstrate a highly transparent laminate electrode for PSCs with stabilized power conversion efficiencies of over 7%. The electrode is composed of a mesh-like silver network on polyethylene terephthalate (PET), coated with a PEDOT:PSS/sorbitol film. Process parameters were optimized for the film thickness of the conductive glue (to planarize the metal mesh), the sorbitol content (for effective adhesion), and the laminate electrode pre-annealing temperature (to remove water from PEDOT:PSS, but not to evaporate sorbitol from the film). The lamination process is simple, compatible with high-throughput processing and avoids the thermal evaporation step of the metal top electrode. Simple manufacturing steps can lead to low-cost, high-efficiency PSC technologies.

## 2. Experimental details

Chemicals were purchased from commercial sources in a high purity grade and were used as received. Methyl ammonium iodide (MAI) was synthesized and purified according to the procedure in [26].

Fluorine doped tin oxide (FTO) glass substrates (TCO22-7 from Solaronix (Aubonne, Switzerland), 7 Ω square^−1^, 2.5 × 2.5 cm^2^) were patterned by etching with Zn powder and HCl (conc.) and were then cleaned (Hellmanex®, (Hellma, Müllheim, Germany), water, acetone, 2-propanol). For the formation of a compact TiO_2_ blocking layer, TiCl_4_ (25 ml) was hydrolyzed in water (90 ml) at 0°C and the resulting solution was spin coated at 5000 rpm for 30 s. A mesoporous TiO_2_ layer was applied by spin coating a paste of TiO_2_ nanoparticles (Ti-Nanoxide T/SP from Solaronix diluted with ethanol 0.22% wt/wt) at 5000 rpm for 30 s followed by gradual heating to 380°C for 10 min and then to 500°C for 30 min in air.

The perovskite layer was prepared on top of mesoporous TiO_2_ inside a nitrogen-filled glovebox following a reported method.[27] A solution of the precursors PbI_2_ (Sigma-Aldrich (St. Louis, MO, United States), 99.999%) and MAI in dimethyl sulfoxide (DMSO, 1:1 M ratio, concentration 45% wt/wt) was spin-coated at 1000 rpm for 10 s and then at 5000 rpm for 30 s. During the last 5 s of spin-coating 1 ml chlorobenzene was dropped on the spinning substrate. The perovskite films were annealed at 100°C for 10 min.

The hole transport material (HTM) 2,2′,7,7′-tetrakis-(N,N-di-p-methoxyphenyl-amine)-9,9′-spirobifluorene (Spiro-OMeTAD, Sigma-Aldrich 99%) with dopant (FK209 from Dyesol (Queanbeyan, Australia)) and additives (4-tert-butylpyridine and lithium bis(trifluoromethylsulfonyl)imide, Sigma-Aldrich) was prepared and deposited as previously described.[28]

Cells were finished by laminating the top electrode on the HTM. For comparison, Au electrodes were deposited on the same substrates. 70 nm of Au (Kurt J. Lesker (Jefferson Hills, PA, United States), 99.999%) was thermally evaporated at < 5 × 10^−6^ mbar through a shadow mask. For lamination, a commercial substrate from Cima Nanotech (Oakdale, MN, United States; SANTE FS200, batch number 140829-4-5) was used. The substrate was washed with isopropanol and water, dried in air and was then treated with oxygen plasma for 3 min. To a PEDOT:PSS dispersion (Clevios F CE, from Heraeus Precious Metals (Leverkusen, Germany), solid content 3 – 4%), D-sorbitol (Sigma-Aldrich, ≥98%) was added in amounts of 0–400 mg ml^−1^ and stirred overnight. This solution was then filtered (5 μm) and spin coated onto the substrate. The thickness of the coated PEDOT:PSS/sorbitol film was adjusted by changing the spin speed. For a sorbitol concentration of 286 mg ml^−1^ (400 mg sorbitol added to 1 ml PEDOT:PSS dispersion), a spin speed of 1000 rpm resulted in a thickness of ~1.3 μm. Indicated thicknesses were measured on reference films that were coated on glass. The PET/Ag-mesh/PEDOT:PSS/sorbitol electrode was then annealed at 120°C for 10 min outside the glove box and was laminated when still hot on the HTM layer of the pre-finished cell using finger pressure. For the lamination process the bottom part of the device was kept at room temperature. The area of the laminated electrode was typically 0.5 × 0.5 cm^2^.

Solar cells were characterized in ambient atmosphere. Current-voltage (JV) characteristics were measured by a Keithley 2400 source/measure unit (Cleveland, Ohio, United States) in the dark and under simulated AM1.5G solar irradiation of 100 mW cm^−2^ from a calibrated solar simulator (Spectra-Nova (Ottawa, Canada)). Cells were masked with a metal aperture of 0.2 cm diameter to define the active area. Current-voltage curves were recorded at a scanning rate of 0.1 V s^−1^. A Cornerstone 130 monochromator (LOT-QuantumDesign (Romanuel-sur-Morges, Switzerland)) was used together with a 300 W Xe lamp to measure the incident photon to current conversion efficiencies (IPCE). The monochromatic light intensity was determined using a calibrated Si-diode.

Cross-sectional images of cells were taken on a SU8200 (Hitachi (Tokyo, Japan)) cold field emission scanning electron microscope (SEM) at a typical acceleration voltage of 1.5 kV and emission current of 5.0 μA. Atomic force microscopy (AFM) experiments were carried out on a NanoSurf Mobile S instrument (Nanosur (Liestal, Switzerland)) in tapping mode. Film thicknesses were determined by profilometry (Ambios XP1 from Ambios Technology, (Santa Cruz, CA, United States)). Optical microscope images were taken with an 3D microscope (Leica DCM8, Leica Microsystems, (Heerbrugg, Switzerland)). Conventional transmission spectra were measured on a Varian Cary 50 UV-vis spectrophotometer (Agilent Technologies, (Santa Clara, CA, United States)). Transmission spectra using an integrating sphere were obtained on a Flurolog-3 from Horiba Jobin Yvon (Bensheim, Germany).

For conductivity measurements three Au or Ag contacts were evaporated on glass/PEDOT:PSS/sorbitol or PET/Ag-mesh/PEDOT:PSS/sorbitol samples with defined distances of 2.6 and 7.8 mm. By measuring the conductivity for different channel lengths the effect of the contact resistance could be separated. Conductivity samples were pre-heated at 120°C for 10 min and measurements were performed in the glovebox. For sorbitol concentrations higher than 286 mg ml^−1^, the conductivity dropped rapidly below the detection limit of the measurement setup.

## 3. Results and discussion

**Figure 1.**
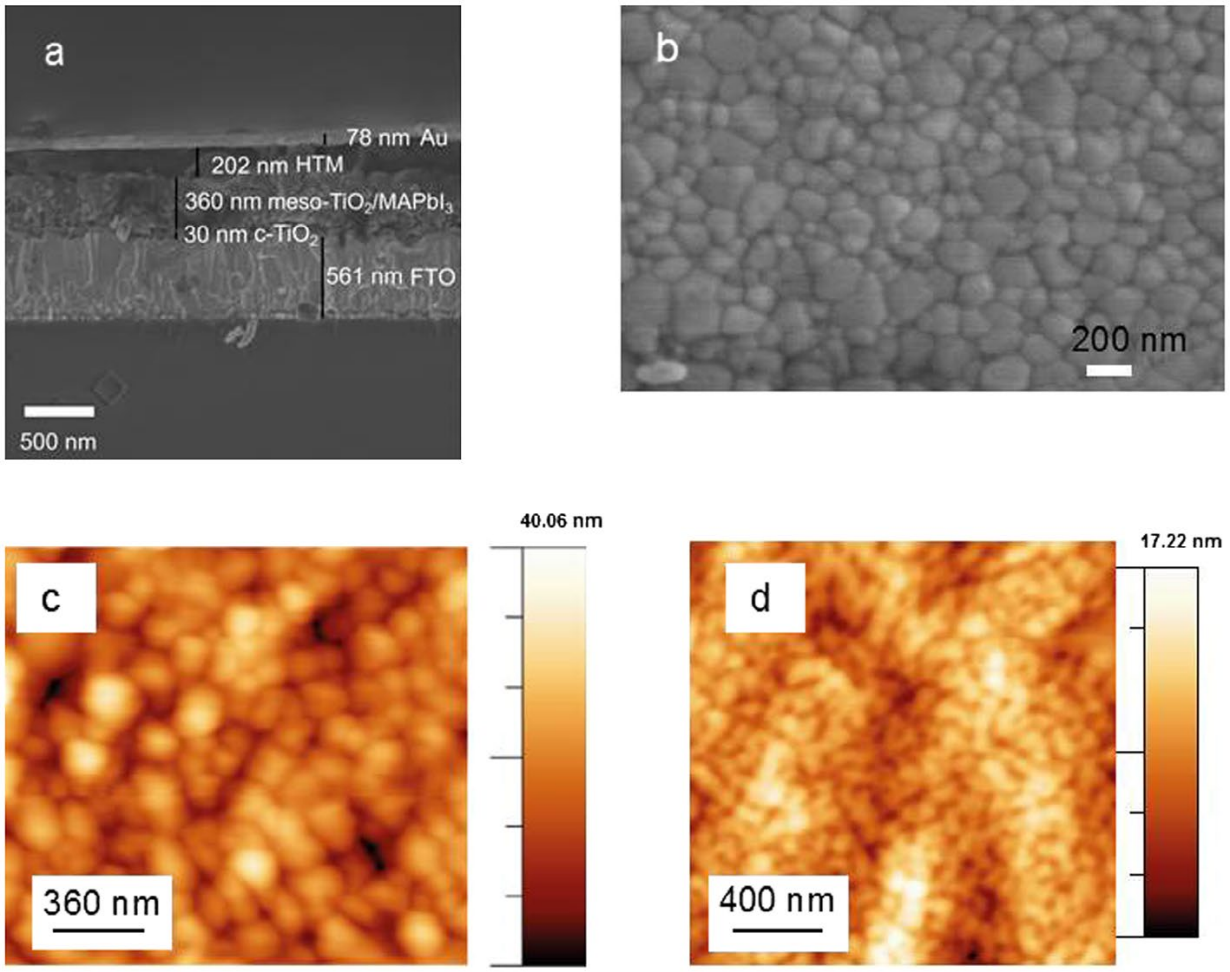
(a) Cross-sectional SEM image of the perovskite solar cell. For laminated devices, gold (Au) was replaced by a PET/Ag-mesh/PEDOT:PSS/sorbitol electrode. (b) Top-view SEM image of the perovskite film. (c) AFM topography of the surface of the perovskite film. (d) AFM topography of the surface of Spiro-OMeTAD coated on the perovskite layer.

Figure 1(a) shows a cross-sectional SEM image of PSCs fabricated in this work. The device consists of a fluorine doped tin oxide layer on glass, a compact TiO_2_ layer, mesoporous TiO_2_ infiltrated with methylammonium lead iodide perovskite (MAPbI_3_) and a HTM (Spiro-OMeTAD). A gold top electrode was evaporated on the HTM layer shown for the cell in Figure 1(a), and gold was replaced by a transparent electrode for laminated devices.

The perovskite layer was prepared by coating an equimolar mixture of PbI_2_ and MAI in DMSO and adding chlorobenzene at the end of the spin-coating process.[27] The top-view SEM image of the perovskite layer in Figure 1(b) indicates a uniform coverage with crystal sizes in the order of 100–200 nm. The film surface topography was characterized with AFM. For the perovskite layer the rms roughness was 9.1 nm, and when coating Spiro-OMeTAD on top the rms roughness decreased to 2.3 nm (Figure 1(c) and (d)). This provides a fairly smooth surface for the lamination process of the top electrode.

**Figure 2.**
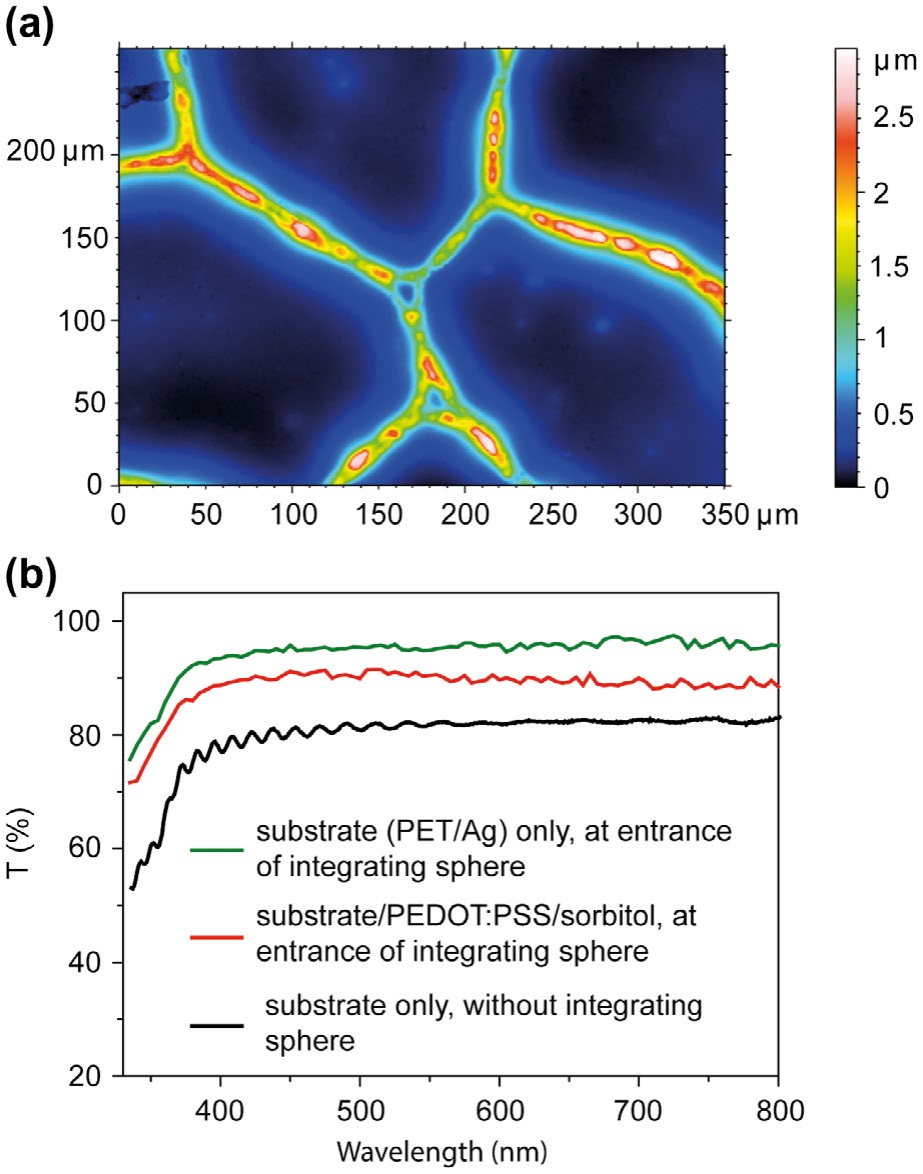
(a) Optical confocal microscopy image of the commercial substrate. The color axis indicates the height distribution of the Ag network on PET. (b) Selected transmission spectra of the electrode substrate and the laminate electrode.

For lamination a commercial, flexible, transparent and conductive substrate was used. The substrate consists of a random mesh-like silver (Ag) network on PET with a geometrical open area of ~90% and a sheet resistance of ~13 Ω square^−1^. The metal linewidths are ~4–12 μm with thicknesses ~0.7–1.3 μm, as determined from profilometer and optical confocal microscopy measurements (Figure 2(a)). The substrate was then coated with PEDOT:PSS dispersions containing varying amounts (0 to ~30 wt%) of sorbitol. The function of PEDOT:PSS/sorbitol is to smoothen and planarize the metal network, to collect and transport the holes to the current-collecting metal, and to promote adhesion to the HTM layer during lamination.

The substrate and the laminate electrode are highly transparent (Figure 2(b)). To detect both the directly transmitted and diffusively scattered light, samples were placed at the entrance of an integrating sphere. For comparison, the transmission spectrum of the substrate was measured on a conventional UV-vis spectrophotometer. The full transmission of the substrate is ~94% for wavelengths above 450 nm. The substrate specular transmission is ~82% when measured on a conventional UV-vis spectrometer. The difference is due to light that is diffusively scattered in the forward direction. We determined the angle dependence of the light scattering. Samples were illuminated and the intensity of the transmitted light was measured at variable angles using a silicon photodiode. We found that 95% of light is transmitted within an aperture angle of ±4°. By comparing measurements at the entrance of and inside the integrating sphere, the fraction of light that is reflected by the metal mesh was determined to ~3.6%. When coating a 1.3 μm thick film composed of PEDOT:PSS with 28.6 wt% sorbitol onto the substrate, the full transmission of ~85% of the electrode remained high (Figure 2(b)). That such a thick PEDOT:PSS/sorbitol layer does not absorb more light is indeed expected in our case, because the weight fraction of PEDOT:PSS in the coated film accounts to less than 10%.

We examined the lamination parameters and studied their effect on the solar cell performance. First, we found it necessary to coat the adhesion layer with a minimal thickness of ~1.3 μm onto the substrate. For PEDOT:PSS/sorbitol films with nominally smaller thicknesses the adhesion was poor, electrodes delaminated immediately when applying a small mechanical stress and no working cells could be fabricated. This observation was independent of the annealing temperature. For small film thicknesses, the metal network is not fully covered by PEDOT:PSS/sorbitol and during lamination the protruding metal makes contact with the HTM of the sub-cell, rather than the adhesive.

For ~1.3 μm thick films, we optimized in a second step the sorbitol content and the temperature during lamination. Annealing of the laminate electrode must be performed within a rather narrow processing window of ~100 to ~120°C. The annealing temperature should be at least 100°C to remove water from the PEDOT:PSS dispersion and to melt sorbitol. On the other hand sorbitol evaporates at ~120°C [29]; consequently, laminate electrodes with 28.6 wt% sorbitol that we pre-heated at 140–150°C for 10 min lost their adhesive properties. We fixed the pre-annealing parameters to 120°C and 10 min but note that slight variations of temperature and time did not significantly influence the lamination quality.

PSCs were fabricated using laminate electrodes with 1.3 μm thick spin-coated adhesion layers. The sorbitol content was varied; electrodes were pre-annealed at 120°C for 10 min and applied when still hot directly onto the Spiro-OMeTAD layer. During lamination, the pre-fabricated PSC was kept at ambient temperature. The open-circuit voltage (V_oc_) was independent of the sorbitol concentration. However, we measured a strong increase in the fill factor (FF, from 39% to over 60%) and the short-circuit current (J_sc_, from 8.4 mA cm^−2^ to 20 mA cm^−2^) when increasing the sorbitol from 9.1 wt% to 28.6 wt%. This indicates that the contact at the HTM/laminate interface improves with increasing adhesive concentration, allowing for better p-type charge collection. The conductivity of PEDOT:PSS/sorbitol films decreased with sorbitol content from (10 ± 2) S cm^−1^ (9.1 wt% sorbitol) to (1.0 ± 0.2) S cm^−1^ (28.6 wt% sorbitol). These conductivity values are enough for vertical charge extraction to the low-resistive metal mesh that ensures horizontal current transport out of the device. We found best-performing PSCs for a sorbitol content of 28.6 wt%. For higher sorbitol fractions FF and J_sc_ values dropped sharply, possibly due to a strong decrease in the film conductivity which was below the detection limit of our experimental setup. Note that the decreasing conductivity with higher sorbitol content is not in conflict with the general understanding that addition of sorbitol increases the film conductivity by several orders of magnitude. It has been shown that the conductivity rise coincides with evaporation of sorbitol from the film, i.e. for temperatures above ~120°C.[29]

**Figure 3.**
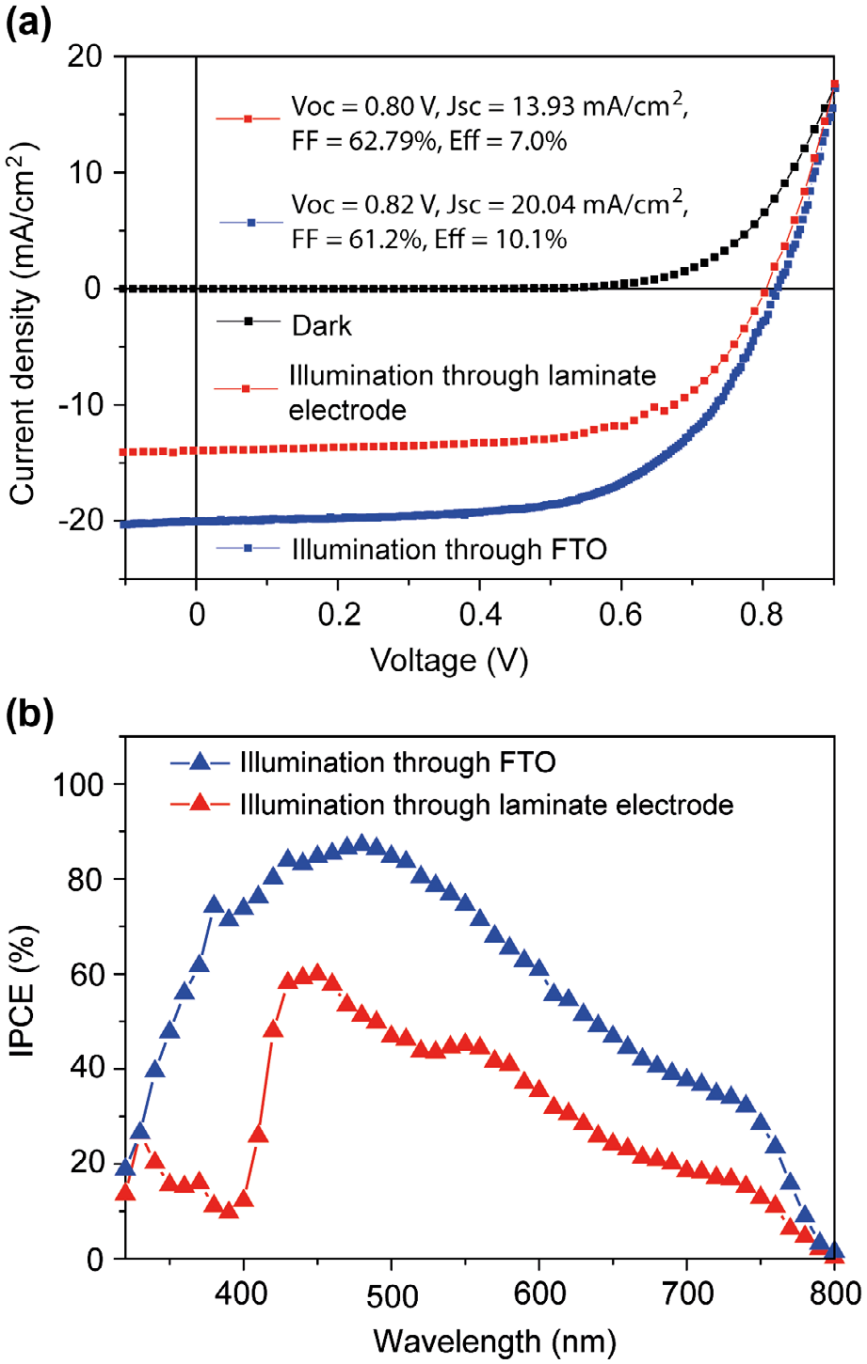
JV-scans (a) and corresponding IPCE curves (b) for the best-performing cell when illuminated through the FTO and the laminate electrode. Cells were scanned from forward bias to short-circuit current (reverse scan) at a scan rate of 0.1 V s^−1^.

Because of the high transmittance of the laminate electrode, cells can be illuminated from both sides. A comparison of JV-scans for the two illumination directions is shown in Figure 3(a); average performance parameters are shown in Table 1. V_oc_ and FF did not depend on the illumination direction, but J_sc_ was higher when illuminating through FTO. The J_sc_ decrease is not due to light absorption of the laminate electrode, because the transparency is over 80% (Figure 2(b)) and comparable to glass/FTO/TiO_2_.[21] One reason is Spiro-OMeTAD, which exhibits a strong absorption in the ultraviolet region. When illuminating through the laminate electrode, light is absorbed by the HTM and does not contribute to current generation. This explains the sharp drop in IPCE observed for wavelengths below ~450 nm (Figure 3(b)). However, IPCE values are smaller over the whole wavelength range of absorption when illuminating through the laminate electrode. This indicates that the overall charge generation and extraction efficiency in our PSCs depends on the position where the light is predominantly absorbed, and that the charge collection is more efficient after light absorption by perovskite material in proximity to TiO_2_, in contrast to the HTM.[12,21,30]

**Table 1.** Average solar cell performance parameters and their accuracies (in round brackets).

	V_oc_ (V)	J_sc_ (mA cm^−2^)	FF (%)	Eff (%)
*Laminate electrode*				
FTO illumination, reverse scan {26} ^a^	0.86 (0.05)	16 (3)	64 (6)	8.8 (1.0)
FTO illumination, reverse scan {5 best of 26}^b^	0.86 (0.04)	17 (2)	66 (4)	9.6 (0.4)
Laminate illumination, reverse scan {9}	0.80 (0.01)	13.7 (0.4)	61 (6)	6.7 (0.6)
FTO illumination forward scan {5}	0.84 (0.07)	12 (3)	60 (2)	6.0 (0.6)
FTO illumination stabilized efficiency {4}				7.6 (1.0)
*Au electrode*				
Reverse scan {26}	0.91 (0.06)	17 (2)	65 (4)	10.1 (1.8)
Reverse scan {5 best of 26}^b)^	0.97 (0.02)	18.9 (0.8)	70 (2)	12.8 (0.7)
Reverse scan {best}	1.00	19.47	71.2	13.9
Forward scan {7}	0.91 (0.03)	17 (2)	58 (8)	9.0 (1.5)
Stabilized efficiency {4}				9.4 (1.5)

^a^ Reverse scan denotes a voltage scan from forward bias to short-circuit current, forward scan from short-circuit current to forward bias. The number of fabricated and measured cells is indicated in curly brackets.

^b^ Performance parameters for a reduced device fabrication yield.

Our PSCs exhibit a hysteresis with sweep direction during JV measurements.[1] JV curves in Figure 3(a) were obtained by scanning from forward bias to J_sc_, and data in Table 1 show that this results in higher average power conversion efficiencies (8.8 ± 1.0%) than when the scan direction is inverted (forward scan, 6.0 ± 0.6%). To estimate the actual efficiency, we measured the current over time for cells that were biased close to their maximum power points. Currents stabilized after ~10 s and the corresponding stabilized average efficiency was (7.6 ± 1.0)%. These power conversion efficiencies were obtained for a device fabrication yield of 100%, meaning that no devices were discarded as low-performing outliers. In our opinion this truly reflects the ability of the lamination process. Clearly, increased performance values can be obtained when selecting a reduced device fabrication yield (Table 1).

Performances for PSCs with a gold top electrode are slightly higher compared to the laminate electrode (Table 1), mainly due to higher J_sc_ and V_oc_. The current increase is likely due to reflection by the Au electrode of non-absorbed light back into the active material, and the slight voltage drop due to a lowered work function of doped PEDOT:PSS, as compared to Au.[31,32]

Studies on the long-term stability of PSCs are ongoing. In preliminary experiments we found for PSCs with a gold electrode and for a storage time of four weeks under inert conditions that V_oc_ and J_sc_ remained almost constant but FF decreased by ~5%. For continuing storage of these cells outside the glovebox in the dark for one week, J_sc_ decreased by ~9% and the performance values reached V_oc_ = (0.9 ± 0.1 V), FF = (65 ± 6%), J_sc_ = (15 ± 3 mA cm^−2^), (three cells, reverse scan). For laminated cells that were stored under nitrogen the mechanical adhesion of the electrode remained intact. Over a storage time of six weeks V_oc_ and FF were constant within ±2%, and J_sc_ decreased by ~9%. However, continuing storage of these laminated cells in ambient conditions induced a substantial decrease of ~20% for V_oc_, J_sc_ and FF. After one week, we measured V_oc_ = (0.75 ± 0.01 V), FF = (49 ± 3%), J_sc_ = (9 ± 2 mA cm^−2^), (three cells, FTO illumination, reverse scan). We ascribe this observation to the hygroscopic nature of PEDOT:PSS, with the implication that laminated PSCs must be carefully encapsulated to prevent the ingress of moisture.

## 4. Conclusions

Our results show that for solution-processed PSCs, lamination of the top electrode provides a low-cost and roll-to-roll compatible alternative to a gold evaporated contact. The concept is applicable to other conductive substrates with, for example, integrated barrier properties against the ingress of water and oxygen. In currently ongoing studies we examine the lamination process to manufacture flexible PSCs. Due to the high transparency of the laminate electrode, flexible devices can be made semitransparent at the same time. Semitransparent PSCs are receiving attention [33,34] because of their specific applications in power-generating windows or greenhouses.

## Disclosure statement

No potential conflict of interest was reported by the authors.
